# Ruptured giant lateral thoracic meningocele associated with intracranial hypotension syndrome in neurofibromatosis type 1: case report with long-term follow-up

**DOI:** 10.3389/fonc.2025.1662107

**Published:** 2025-11-21

**Authors:** Wenfu Li, Hui Zhou

**Affiliations:** 1 Department of Radiology, Affiliated Hospital of Zunyi Medical University, Medical Imaging Center of Guizhou Province, Zunyi, Guizhou, China; 2 Department of Critical Care Medicine, Affiliated Hospital of Zunyi Medical University, Zunyi, Guizhou, China; 3 Department of Graduate School, Zunyi Medical University, Zunyi, Guizhou, China; 4 Department of Radiology, People’s Hospital of Honghuagang District, Zunyi, Guizhou, China

**Keywords:** meningocele, intracranial hypotension syndrome, neurofibromatosis type 1, magnetic resonance imaging, case report

## Abstract

Lateral thoracic meningocele is a rare clinical entity typically associated with neurofibromatosis type 1 (NF1), which can progressively enlarge under the cerebrospinal fluid (CSF) pressure. Despite its rarity, these lesions may rupture or be associated with intracranial hypotension syndrome (IHS). We herein describe a 50-year-old woman presenting with a 2-year history of dyspnea, positional headaches, and dizziness. Imaging revealed a giant lateral thoracic meningocele with right-sided pleural effusion. Following thoracentesis with evacuation of 480 ml effusion (later confirmed as CSF), her headache intensified markedly; subsequent brain MRI demonstrated characteristic features of IHS. Surgical excision of the meningocele was performed, and the patient experienced a favorable recovery, with discharge on postoperative day 22. This case underscores critical clinical insights: NF1-associated giant lateral thoracic meningoceles may manifest respiratory symptoms alongside severe complications, including rupture and IHS. Crucially, procedures such as thoracentesis require extreme caution, as they risk exacerbating CSF leakage and may precipitate life-threatening cerebellar tonsillar herniation.

## Introduction

NF1 is a multisystem neurocutaneous disorder with an incidence of 1 in 3000 live births (1). Diagnostic clinical manifestations for NF1 include café-au-lait macules, axillary or inguinal freckling, iris hamartomas, neurofibromas, optic pathway gliomas, and developmental skeletal abnormalities, particularly sphenoid dysplasia (2). However, there are many other manifestations of NF1, such as interstitial lung disease, vascular dysplasia, and spinal deformities. Scoliosis is the most common spinal abnormality in patients with NF1, with an incidence rate ranging from 10% to 30% (3). Dural ectasis and meningocele are uncommon manifestations, but those significantly associated with NF1 occur in up to 70%-80% of cases and are usually asymptomatic (2, 4). Notably, lateral thoracic meningocele can gradually enlarge under the CSF pressure and pulsations, resulting in compression of adjacent lung tissue, and causing shortness of breath or dyspnea (5). Once the lateral thoracic meningocele has ruptured, thoracentesis and drainage can be fatal if acute cerebellar tonsil herniation occurs. Thus, this case report aims to deepen the knowledge of the relationship between NF1, meningocele, and IHS. Such characteristics will be vital for clinicians in developing safe and effective therapeutic strategies.

## Case description

A 50-year-old female patient was admitted to our Department of Respiratory and Critical Care Medicine on April 11, 2023, for evaluation of a 2-year history of shortness of breath. Over the past few years, she has also frequently experienced headaches and dizziness. For the 20 days prior to admission, she had a persistent headache that worsened in the upright position. She reported no cough, sputum production, nausea, vomiting, or other discomfort. Physical examination revealed diffuse hyperpigmentation over the entire body, along with multiple verrucous skin projections, as well as scoliosis and kyphosis deformities. The patient had been evaluated at our hospital 8 years ago and diagnosed with a “benign spinal tumor,” but she did not take it seriously and received no treatment. Two years ago, she underwent abdominal cyst excision at an outside hospital (specific surgical details and pathology unknown). She denied any history of chronic diseases such as hypertension or diabetes, as well as infectious diseases like hepatitis B or tuberculosis. Admission laboratory tests showed: Complete Blood Count (CBC): Elevated white blood cell count at 14.29 ×10^9^L (reference range: 3.5-9.5 ×10^9^/L) and elevated absolute neutrophil count at 11.29 ×10^9^/L (reference range: 1.8-6.3 ×10^9^/L); other parameters were within normal limits. Electrocardiogram (ECG) and echocardiogram showed no significant abnormalities.

**Figure 1:**
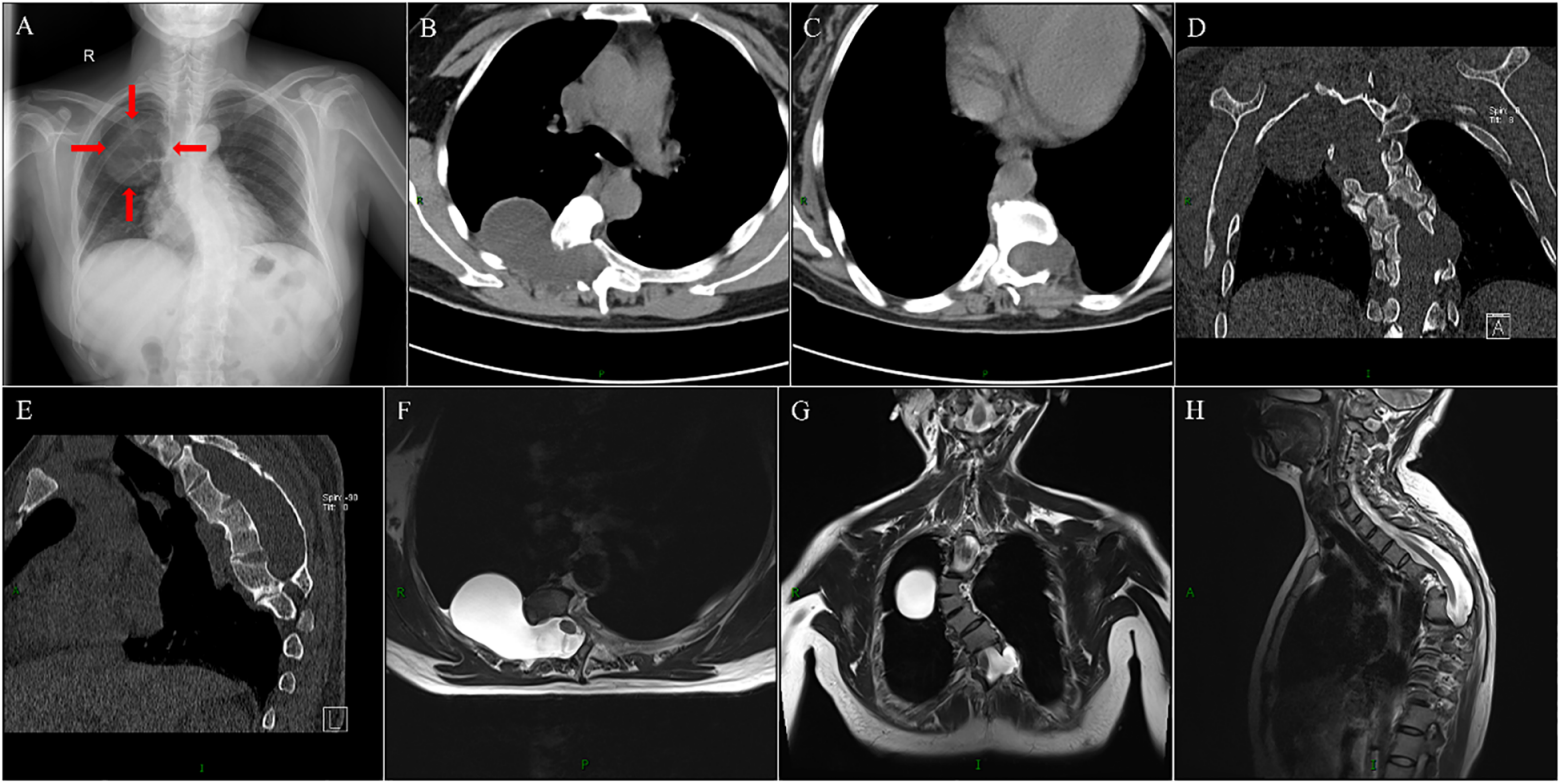
Imaging findings from April 2015. Chest X-ray demonstrates an “S”-shaped scoliosis deformity and a space-occupying lesion (later confirmed as meningocele) in the right upper-middle lung field **(A)**. Thoracic spine CT reveals enlargement of the right T5/T6 and left T9/T10 intervertebral foramina, with meningoceles protruding into the thoracic cavity **(B-E)**. Additionally, thoracic spine CT shows fusion deformity of T4-T9 vertebral bodies, vertebral body bone thinned due to compression, and spinal canal enlargement **(B-E)**. On thoracic spine MRI T2-weighted images (T2WI), meningoceles within the right T5/T6 and left T9/T10 intervertebral foramina appear homogeneously hyperintense with clear boundaries, communicating with the dilated thecal sac **(F-H)**.

**Figure 2:**
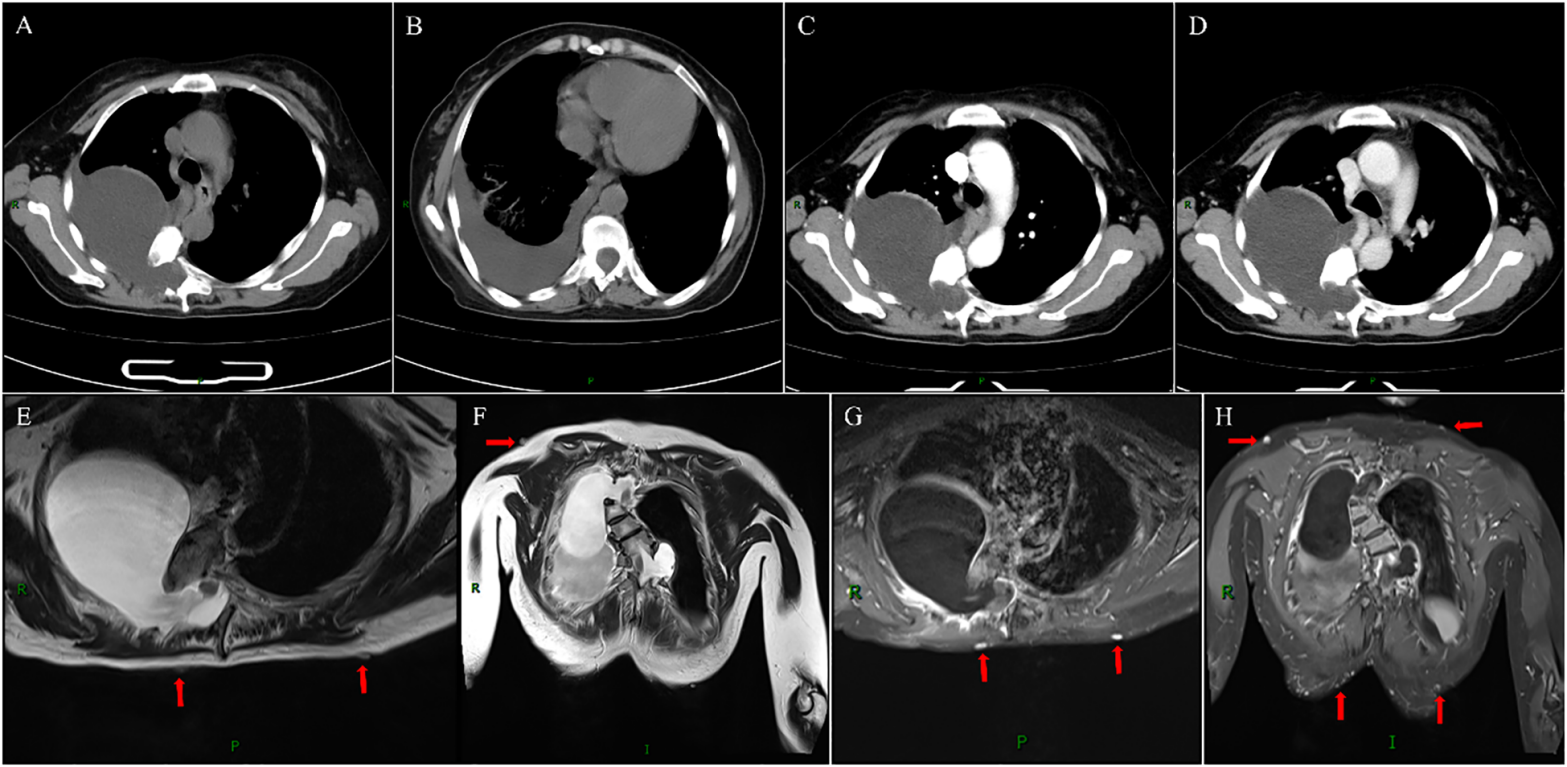
Imaging findings from April 2023. Non-contrast and contrast-enhanced chest CT demonstrates significant enlargement of the right T5/T6 foraminal meningocele compared to 2015, with no internal enhancement. New right-sided pleural effusion is present **(A-D)**. Post-thoracentesis thoracic spine MRI (non-contrast + contrast) reveals a homogeneous T2-hyperintense meningocele at the right T5/T6 foramen. Contrast-enhanced sequences show no internal enhancement of the lesion but prominent posterior wall enhancement, potentially related to the puncture procedure. Additionally, multiple enhancing cutaneous and subcutaneous nodules are noted on MRI (red arrows) **(E-H)**.

To clarify the diagnosis, we retrieved the patient’s April 2015 electronic medical records and imaging data via the Hospital Information System (HIS) and Picture Archiving and Communication System (PACS): Eight years ago, she presented to our hospital after an incidental finding of an intraspinal space-occupying lesion during a physical examination, undergoing chest/thoracic spine X-ray, thoracic Computed Tomography (CT), and MRI. chest/thoracic spine X-ray revealed S-shaped scoliosis with kyphosis and a large space-occupying lesion in the right upper-middle lung field (**Figure 1A**); thoracic CT and MRI further demonstrated abnormal T4-T9 vertebral morphology, T8/T9 intervertebral space narrowing, and enlarged intervertebral foramina at right T5/T6, T6/T7 and left T9/T10 levels with corresponding lateral thoracic meningoceles—notably, a larger right T5/T6 meningocele mildly compressed adjacent lung tissue (**Figures 1B–H**). Conservative management was chosen given her asymptomatic status and personal/family preference. Two years ago, she gradually developed shortness of breath and dizziness but neglected these symptoms and received no standardized treatment until returning to our hospital in April 2023 for further management; non-contrast and contrast-enhanced chest CT showed significant enlargement of bilateral thoracic meningoceles compared to 8 years prior (2015), particularly at the right T5/T6 foramen, accompanied by pleural effusion (**Figures 2A–D**).

Based on the characteristic clinical findings of multiple café-au-lait spots (as evidenced by diffuse hyperpigmentation), axillary or inguinal freckling, multiple cutaneous neurofibromas (verrucous projections), and distinctive skeletal abnormalities (scoliosis and kyphosis) accompanied by bilateral lateral thoracic meningoceles, the patient was diagnosed with NF1 during this admission according to the National Institutes of Health (NIH) diagnostic criteria (6).

**Figure 3:**
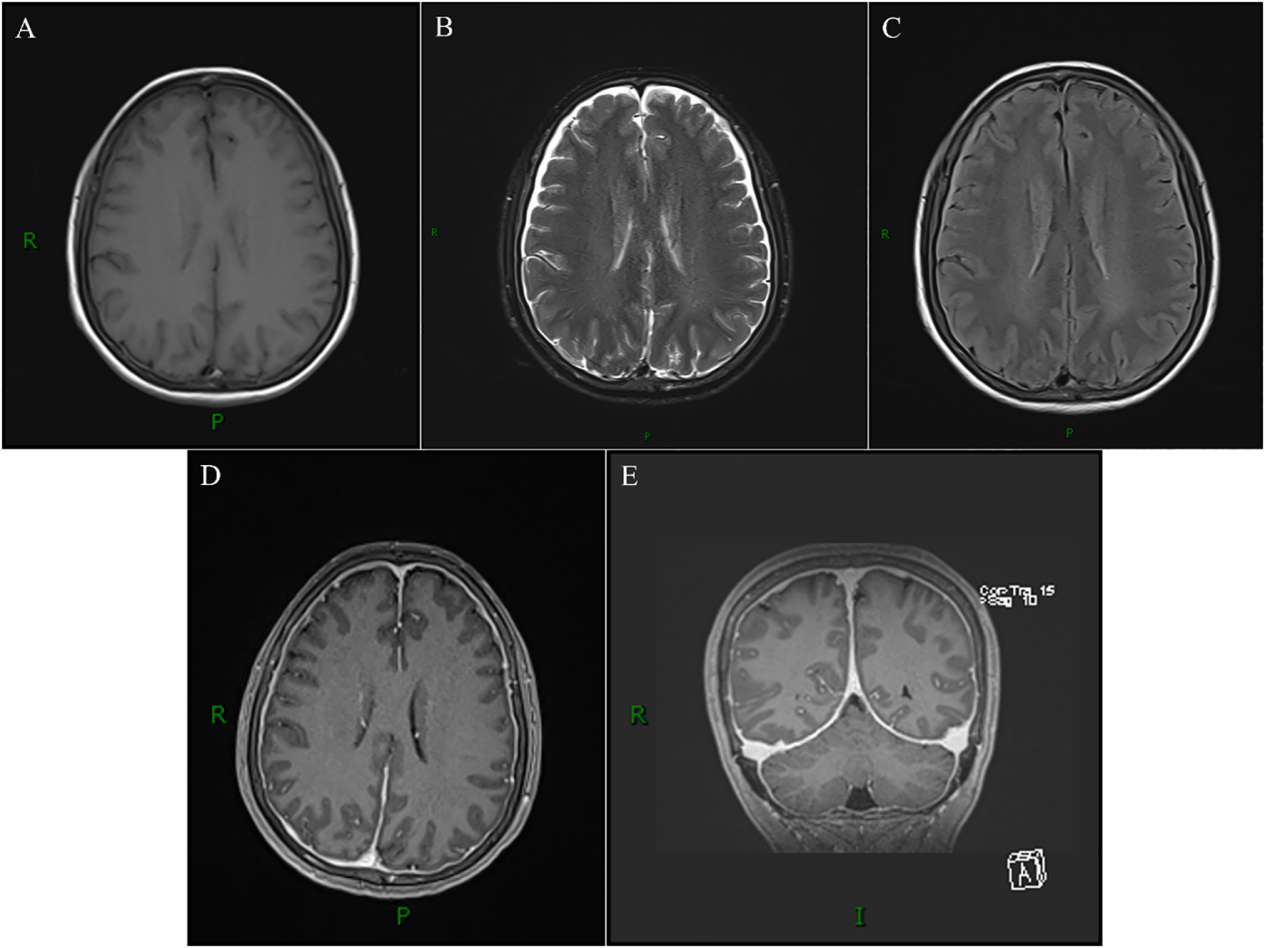
Post-thoracentesis brain MRI (non-contrast + contrast) from April 2023 demonstrates bilateral subdural fluid collections **(B)**, diffuse pachymeningeal thickening with enhancement **(D, E)**, and dilation of bilateral transverse sinuses **(E)**.

After admission, diagnostic and therapeutic thoracocentesis was performed. A total of 480 ml of clear, colorless pleural fluid was aspirated in two sessions and sent for routine and biochemical analysis, later confirmed as CSF. Following the second aspiration, the patient’s headache progressively intensified and persisted independently of positional changes. Subsequently, non-contrast and contrast-enhanced MRI of the thoracic spine and brain were conducted. Post-thoracentesis thoracic spine MRI (T_2_WI and contrast-enhanced T_1_WI) revealed new prominent posterior wall enhancement of the right T5/T6 meningocele compared to pre-thoracentesis CT, along with multiple enhancing cutaneous/subcutaneous nodules at the corresponding thoracic level (**Figures 2E–H**). Brain MRI revealed bilateral subdural fluid collections, significant dural thickening with enhancement, and marked dilation of dural venous sinuses—findings consistent with IHS (**Figure 3**). Additionally, contrast-enhanced MRI demonstrated multiple enhancing cutaneous nodules. Based on characteristic clinical symptoms and imaging features, the patient was diagnosed with NF1 complicated by IHS secondary to ruptured giant lateral thoracic meningoceles and was referred to neurosurgery for further management.

Despite fluid supplementation and strict supine positioning support, the patient’s headache persisted. Attributing symptom exacerbation to accelerated CSF leakage from thoracocentesis, neurosurgical intervention was performed. Intraoperative findings included T4-T7 vertebral fusion, segmental laminar defects, and kyphoscoliosis deformity. Exploration revealed a giant right T4-T6 foraminal meningocele herniating into the thoracic cavity, containing copious CSF. Following partial excision of the cyst wall, the residual wall was inverted and sutured under microscopic guidance to achieve a watertight dural closure. This repair was then reinforced with a synthetic dural graft sutured over the defect. Finally, an autologous muscle flap was placed over the graft to further safeguard against cerebrospinal fluid leakage. Titanium mesh was implanted externally to prevent recurrence. Concurrently, the left T9/T10 meningocele was reduced and repaired, with T3-T9 pedicle screw fixation. Given the extensive laminectomy defects, pre-existing spinal instability due to kyphoscoliosis, and the need to protect the dural repair from mechanical stress, posterior spinal instrumentation and fusion from T3 to T9 were performed using pedicle screw fixation. Due to intraoperative blood loss (1,000 ml), the patient received supportive care in the Department of Critical Care Medicine and was discharged on postoperative day 22.

**Figure 4:**
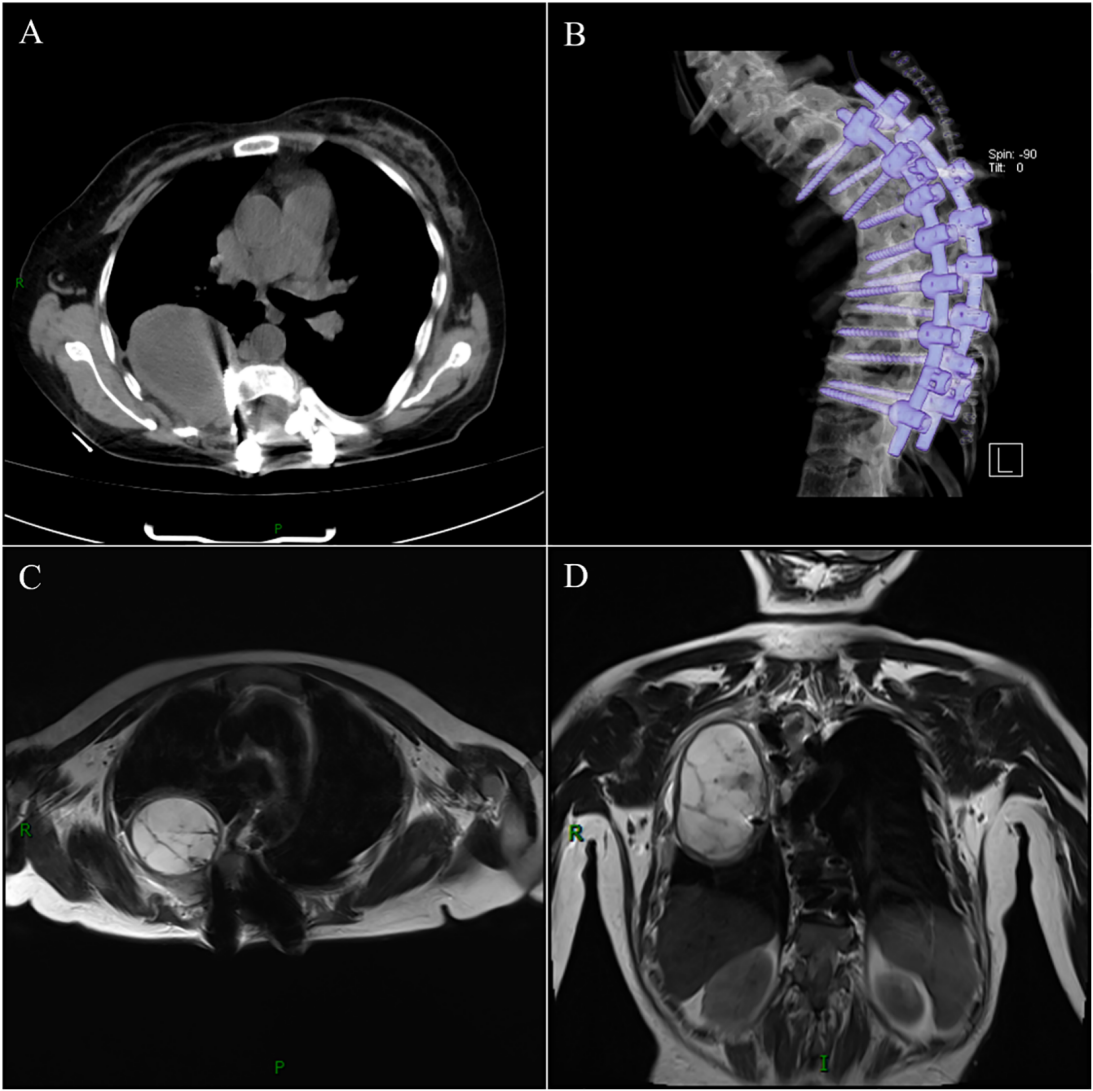
Initial postoperative chest CT: Demonstrates a residual cystic lesion in the right thoracic cavity at T5/T6 level with significant volume reduction compared to preoperative scans. Note well-positioned posterior titanium mesh grafting and T3-T9 pedicle screw fixation **(A, B)**. Thoracic MRI at 3-month follow-up: Confirms stable size of the residual cystic lesion without interval growth and absence of recurrent pleural effusion **(C, D)**.

Initial postoperative chest CT demonstrated a cystic lesion mass in the right thoracic cavity at T5/T6 level, showing significant volume reduction compared to preoperative imaging, with complete resolution of right pleural effusion (**Figures 4A, B**). At the 3-month follow-up, thoracic MRI confirmed stabilization of the cystic lesion mass without interval growth versus initial postoperative CT and absence of pleural effusion recurrence (**Figures 4C, D**). Concurrently, the patient reported marked improvement in dyspnea and orthostatic headache. During the 26-month follow-up as of manuscript submission, symptoms remained resolved without new neurological or respiratory complaints.

## Discussion

Neurofibromatosis type 1 (NF1) is an autosomal dominant neurocutaneous disorder resulting from pathogenic variants in the NF1 gene (7). Although the diagnostic criteria established by the National Institutes of Health (NIH) in 1987 remain widely applied in clinical settings, the International Neurofibromatosis Diagnosis Criteria Group (I-NF-DC) introduced revisions in 2021 that formally integrated genetic testing into the diagnostic framework (6, 8). The updated criteria encompass seven cardinal features: six or more café-au-lait macules; two or more neurofibromas of any type or one plexiform neurofibroma; axillary or inguinal freckling; optic pathway glioma; two or more Lisch nodules; characteristic bony lesions; or identification of a heterozygous pathogenic NF1 variant through genetic analysis. Diagnosis is confirmed in individuals without a parental history of NF1 by the presence of two or more of these features, whereas only one feature is required in cases with an affected parent.

The diagnosis of NF1 in our patient was confidently established according to the well-validated NIH diagnostic criteria, based on the presence of several cardinal clinical features. She exhibited classic cutaneous manifestations, including multiple café-au-lait spots, axillary or inguinal freckling, and multiple cutaneous neurofibromas (as evidenced by the verrucous projections on physical examination and enhancing cutaneous nodules on MRI). In contrast, other diagnostic features such as optic pathway glioma, sphenoid wing dysplasia, and Lisch nodules—the latter not assessed by slit-lamp examination in this instance—were not observed. No radiological or intraoperative findings suggested the presence of schwannomas. A definitive family history could not be obtained despite repeated attempts. It is noteworthy that the 2021 I-NF-DC has updated the diagnostic framework to include the detection of a heterozygous pathogenic NF1 variant as a formal criterion. While genetic testing offers definitive confirmation, particularly in phenotypically equivocal cases, the clinical presentation in our patient was sufficiently characteristic to establish the diagnosis based on longstanding NIH criteria, which continue to serve as a diagnostic cornerstone in routine clinical practice. Thus, genetic testing was not pursued as the diagnosis was unequivocal based on clinical grounds.

Spinal deformities are common in NF1, occurring in approximately 10% of patients (9). NF1-related spinal abnormalities involve both bone and soft tissue. Bony changes include scoliosis and vertebral centrum abnormalities, while soft tissue changes encompass dural ectasia, meningoceles, and spinal tumors (10). The appropriate development and upkeep of bone tissue necessitate a well-regulated balance between osteoclasts and osteoblasts. Osteoblasts with an NF1 gene deficiency promote the migration and activation of osteoclasts through cytokines, leading to a dysfunctional cycle of bone formation and destruction, which can result in skeletal abnormalities (7). Dural ectasia is characterized by an enlargement or bulging of the dural sac, which can manifest as meningoceles through enlarged intervertebral foramina or vertebral body osteolysis. These abnormalities are commonly thought to be associated with mesodermal dysplasia, although the precise pathogenesis of this condition remains unclear (11, 12).

Meningoceles are uncommon clinical entities, with the majority occurring post-laminectomy, being classified as pseudo-meningoceles. Congenital meningoceles are rare and typically associated with disorders such as NF1 and Marfan syndrome (13). The thoracic region is the most common site for meningoceles arising in association with NF1, as documented in the medical literature. Thoracic meningoceles usually manifest laterally along the convex aspect of the scoliotic curve (5). This localization is attributed to the heightened pressure gradient between the CSF and the extra-pleural space, which, coupled with the reduced resistance of the paravertebral muscles, predisposes the meninges to herniation (11, 13). Symptoms of meningoceles depend on the size and location of the lesions, which can lead to neurological or respiratory symptoms such as pain, paraparesis, cough, and dyspnea due to compression (14), as observed in our case. However, most patients remain asymptomatic. Spontaneous rupture leading to hydrothorax, hemothorax, or IHS is a rare but critical complication of lateral thoracic meningoceles (4, 15, 16).

IHS, caused by various etiologies, is diagnosed through low lumbar puncture opening pressure (<60 mm CSF) and/or typical imaging signs of intracranial hypotension, manifesting as a clinical syndrome characterized by orthostatic headaches (17, 18). However, low opening pressure is an unreliable diagnostic marker for this disease, as only a third of individuals with confirmed IHS (19). While orthostatic headaches, once regarded as classic and cardinal symptoms of IHS, are not always present (17). Therefore, although IHS is not an uncommon disorder, it is frequently misdiagnosed and underdiagnosed, with its etiology not fully understood and often remaining undetermined. Based on etiology, it can be classified into spontaneous and secondary forms. Spontaneous IHS may be related to meningeal structural defects and mechanical factors, such as meningeal diverticula, connective tissue disorders (like Marfan syndrome), and spinal pathologies (including osteophytes, disc herniation, etc.) (20). Secondary IHS is more commonly observed after lumbar puncture, cranial trauma, or postoperatively, and is rarely seen in conditions such as shock, heart failure, and uremia (21). Meningoceles associated with IHS in NF1 are exceedingly rare in clinical practice, with only a limited number of cases documented in the medical literature (4, 5, 22, 23). Huang and colleagues (4) reported a rare case of NF1-related meningocele, initially misdiagnosed as encapsulated pleural effusion, which led to IHS after thoracentesis. Furthermore, unruptured meningoceles in NF1 patients can also cause IHS (5). This rare condition presents a unique set of challenges for both diagnosis and treatment, often requiring a multidisciplinary approach to manage effectively.

It is imperative to recognize that intracranial hypotension secondary to CSF leakage can progress beyond typical IHS to a more acute and lethal entity known as pseudohypoxic brain swelling (PHBS). First systematically described by Van Roost et al. in 2003, PHBS is characterized by rapid neurological deterioration, diffuse cerebral edema on imaging—often with bilateral basal ganglia and thalamic involvement—and clinical signs such as impaired consciousness, seizures, and even circulatory collapse (24). The pathophysiology is thought to involve rapid CSF depletion leading to intracranial hypotension, venous engorgement, and impaired cerebral venous return, resulting in a hypoxia-mimicking pattern of injury without true hypoxia (24–28).

Notably, PHBS has been reported following both cranial and spinal procedures, particularly in the setting of subgaleal or epidural suction drainage, which can precipitate rapid CSF loss and negative intracranial pressure (24, 25, 28). In some cases, such as those reported by Moon et al. and Sviri, PHBS has been associated with bradycardia, hypotension, and cardiac arrest shortly after initiation of suction drainage (25, 28). Importantly, PHBS can occur even in the absence of documented durotomy, as illustrated by Chidambaram et al. in a patient undergoing lumbar decompression and fusion (27).

In the context of NF1 with giant lateral thoracic meningoceles, as in our patient, the risk of PHBS is theoretically elevated due to the large-volume CSF reservoir and potential for rapid egress following rupture or iatrogenic intervention. Although our patient did not develop full-blown PHBS, her acute clinical deterioration following thoracentesis—marked by worsening headache and imaging evidence of IHS—highlights the precarious balance of CSF dynamics in such cases. Thoracentesis or any procedural drainage in these patients should be considered high-risk, as it may accelerate CSF loss and precipitate life-threatening cerebral edema or herniation.

Early recognition of PHBS is critical. Management includes immediate cessation of suction drainage, aggressive ICP management, and in severe cases, surgical decompression. A high index of suspicion should be maintained in any NF1 patient with meningoceles who presents with acute neurological decline following spinal or thoracic intervention.

Imaging studies, especially brain MRI and multimodal spinal imaging, are essential in confirming this disease and excluding other disorders. Imaging characteristics of IHS on cranial MRI, both with and without contrast, are related to CSF volume reduction. They include diffuse gadolinium pachymeningeal enhancement, subdural fluid collections, engorgement of venous structures, pituitary hyperemia, and brain sagging (29). Among these features, diffuse dural thickening and enhancement were the most common indications for IHS, observed in 73% of the 2078 patients (17). This may involve the tearing of small, dilated vessels within the dura mater, which is associated with brain sagging, or it could result from meningeal blood vessel dilation to compensate for CSF depletion. The latter condition, dural hyperemia, also explains the dilation of the dural venous sinuses and the enlargement of the pituitary gland (30). Subdural collections, typically located over the cerebral convexities, can be either unilateral or bilateral and often do not have a mass effect (31). However, in a few cases, subdural hematomas from ruptured bridging veins may cause compression and shift of the brain (30). CSF provides not only mechanical protection for the brain but also buoyancy, effectively reducing its net weight (32). Thus, in patients with IHS, the buoyancy provided by the CSF is reduced, which may lead to brain sagging. Midsagittal MRI represents the optimal approach for assessing brain sagging, as it provides detailed insights into the position of the brain and intracranial structures.

A variety of imaging methods are currently utilized for spinal diagnostic assessments, each possessing its own set of advantages and limitations. X-ray imaging, celebrated for its high spatial resolution, is a common tool in the assessment of spinal scoliosis, adept at delineating macroscopic deformities. However, it faces intrinsic limitations when tasked with discerning the subtle changes within the vertebral bone architecture. CT imaging is renowned for its ability to not only reveal detailed changes in osseous structures but also to offer three-dimensional reconstructions, allowing for multi-angled observation and analysis (2). MRI provides a distinct advantage over X-ray and CT scanning with its superior soft tissue contrast resolution and lack of ionizing radiation, which makes it the modality of choice for comprehensive spinal evaluations. Its application is particularly crucial in the diagnostic workup of patients with suspected IHS, facilitating the precise identification of the underlying causes (29).

The surgical management of lateral thoracic meningoceles remains controversial and poses significant challenges. Direct resection techniques, such as multilayer reinforced repair, can alleviate mass effect but carry risks of CSF leakage and recurrence, closely associated with tissue fragility due to dural dysplasia (11, 33). Cystoperitoneal shunting may necessitate reoperation due to shunt malfunction or worsening intracranial hypotension (5). While programmable shunting (e.g., Strata valves) can balance pulmonary decompression with intracranial pressure (ICP) regulation, it carries risks of device-related complications (5, 34). Recent studies further highlight divergences in treatment strategies: some scholars advocate prioritizing minimally invasive shunting to reduce surgical trauma, while other literature indicates that shunting alone may fail to address structural dural defects, particularly in ruptured lesions. Conversely, open repair directly addresses anatomical abnormalities, yet concerns persist regarding its procedural complexity and postoperative complications (11, 35). This controversy stems from lesion heterogeneity and the multifaceted goals of treatment. For patients with predominant respiratory impairment, surgery must prioritize restoring pulmonary ventilation. When concurrent IHS is present, CSF dynamic disturbances must also be resolved. Current evidence suggests that selecting an appropriate surgical approach requires comprehensive consideration of multiple factors, including rupture status, dural integrity, spinal stability, and the presence of neural compression. Based on available evidence, unruptured cases may be candidates for programmable shunting, whereas ruptured lesions tend to favor multilayer repair, with vigilant postoperative monitoring for recurrence and CSF leakage. Notably, a multidisciplinary approach (e.g., collaboration among neurosurgery, thoracic surgery, and plastic surgery) has been proposed as a strategy for complex cases, though its definitive value requires further validation through research (35).

The role of spinal instrumentation in the repair of lateral thoracic meningoceles warrants specific discussion. In the present case, the primary indication for T3-T9 instrumentation was not the aggressive correction of scoliosis, but rather the restoration of spinal stability following multi-level meningocele resection and dural repair. The advantages of this approach are several: (1) it provides immediate rigid stabilization, protecting the tenuous dural repair from dynamic CSF pressures and minimizing the risk of postoperative CSF leakage or recurrence (11, 33); (2) it mitigates the risk of progressive deformity in a spine already compromised by NF1-associated dysplastic changes (9, 10); and (3) it promotes successful long-term arthrodesis, especially after extensive bony dissection. However, the disadvantages must also be acknowledged. Extensive instrumentation significantly increases surgical complexity, operative time, blood loss, and cost. Furthermore, it carries inherent risks such as screw malposition with potential neurological injury, implant failure, and the long-term possibility of adjacent segment disease (9, 36). Therefore, the decision to perform instrumentation should be individualized, weighing the need for stability against the increased surgical burden.

In conclusion, giant lateral thoracic meningoceles associated with NF1 may precipitate IHS, rendering thoracentesis contraindicated due to accelerated CSF loss. Diagnosis relies on characteristic brain MRI findings (e.g., dural enhancement, brain sagging) and spinal imaging evaluation. Surgical management requires individualized strategies: multi-layer repair is preferred for ruptured cases, while programmable shunting is optimal for unruptured lesions. This case highlights the necessity for heightened clinical vigilance toward this rare NF1 complication and underscores the essential role of multimodal imaging guidance and multidisciplinary collaboration.

Moreover, clinicians should be vigilant for the potential development of PHBS—a life-threatening complication of CSF leakage—in NF1 patients with meningoceles, particularly following diagnostic or therapeutic interventions that may exacerbate CSF loss.

## Data Availability

The datasets presented in this article are not readily available because of ethical and privacy restrictions. Requests to access the datasets should be directed to the corresponding author.
